# Comparison of 3-year outcomes of photodynamic therapy combined with intravitreal ranibizumab or aflibercept for polypoidal choroidal vasculopathy in a European cohort

**DOI:** 10.1007/s00417-022-05724-4

**Published:** 2022-06-09

**Authors:** Siyin Liu, Ramandeep Chhabra

**Affiliations:** 1 Manchester Royal Eye Hospital, Manchester University NHS Foundation Trust, Manchester, UK; 2 School of Biological Sciences, Faculty of Biology, Medicine and Health, University of Manchester, Manchester, UK; 3 Manchester Academic Health Science Centre, Manchester, UK

**Keywords:** Polypoidal choroidal vasculopathy, Ranibizumab, Aflibercept, Photodynamic therapy, Switching

## Abstract

**Purpose:**

Combined use of photodynamic therapy (PDT) with intravitreal anti-vascular endothelial growth factors (anti-VEGF) agents, such as ranibizumab (IVR) or aflibercept (IVA), has been shown to be effective for treating polypoidal choroidal vasculopathy (PCV). However, it is currently not well established which anti-VEGF agent provides superior outcomes for performing combination therapy. The present study compares the visual outcomes and re-treatment burden of combination therapy of PDT with either IVR or IVA in a European cohort of patients with PCV.

**Methods:**

A retrospective analysis was done on PCV patients who had received combination therapy of PDT with either IVR or IVA. The demographic characteristics, visual outcome, and anti-VEGF re-treatment exposures were analysed and compared.

**Results:**

A total of forty-four eyes (*n* = 11 male, 25%) were included in the analysis: 7 patients received IVR, 19 started with IVR but switched to IVA (IVS), and 18 received IVA, in combination with PDT. The BCVA improved in all three groups at 6-, 12-, 18-, 24-, 30-, and 36-month follow-ups after PDT, although the improvement was not statistically significant in the IVR group. The number of intravitreal anti-VEGF injections required/year after PDT was significantly fewer than before PDT. Significantly less eyes in the IVS group attained a good visual acuity of more than 70 ETDRS letters at the final visit.

**Conclusion:**

Both IVR and IVA combined with PDT were effective treatments for the European cohort of patients with PCV. In eyes refractory to IVR, performing PDT promptly may be more beneficial than switching to IVA.


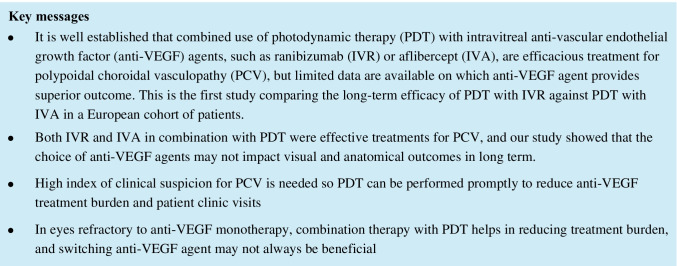


## Introduction

Polypoidal choroidal vasculopathy (PCV) is an exudative maculopathy characterised by the polypoidal dilation with or without branching vascular network of choroidal vessels. Although once considered a variant of neovascular age-related macular degeneration (n-AMD), PCV tends to present in younger patients associated with a different set of clinical features [1]. The prevalence of PCV has been reported to be higher in East Asians than Caucasians [2–7], accounting 22.3–61.3% of patients with suspected n-AMD [3, 4]. However, emerging evidences suggest that the prevalence of PCV in Caucasian is probably underestimated [8].

Photodynamic therapy with verteporfin (PDT) is the first approved treatment modality for n-AMD, including PCV, and it has been widely reported as a successful therapy in inducing occlusion of polypoidal lesions [9, 10]. Intravitreal injection of anti-vascular endothelial growth factor (VEGF), including ranibizumab (IVR) and aflibercept (IVA), has been shown to deliver favourable visual outcome in patients with PCV [11]. Large clinical trials revealed the superiority of intravitreal injections of anti-VEGFs in maintaining vision over PDT monotherapy [12, 13], whilst PDT was more effective in regressing polyps [9] than anti-VEGF monotherapy. Combining the effect of polyp occlusion induced by PDT and maintenance of good vision brought by anti-VEGF is thought to synergistically deliver greater functional and anatomical improvements. The EVEREST-II study established that combination therapy of PDT with adjunct IVR was able to produce superior outcomes in visual gains and polyp regression with less injection required, compared to monotherapy [14]. Multiple studies also concluded that combination therapy of PDT with either IVR or IVA could deliver favourable visual outcomes [15–20].

Whilst evidence supports the combined use of PDT and anti-VEGF agents for treating PCV. It is currently not well established which agent provides a superior outcome for performing combination therapy. The limited existing comparative analyses demonstrated mixed results in the East Asian population [16, 21, 22]. The present study aims to compare the 3-year visual outcomes and re-treatment burden of combination therapy of PDT with either IVR or IVA in a European cohort of patients with PCV.

## Methods

### Study design and population

A retrospective chart review was performed on patients with PCV that received verteporfin PDT at Manchester Royal Eye Hospital (MREH) between January 2016 to January 2019. MREH is a large university hospital and tertiary referral centre for macular diseases. Patient data were extracted from electronic medical records (Medisoft, Leeds, and the UK), and all patient identifiable information were anonymised. Each eye was analysed individually, regardless of the treatment received in the other eye. All the procedures being performed were part of the routine care. The study was approved by the clinical audit department of the Manchester Royal Eye Hospital and conformed to the standards described in the Declaration of Helsinki.

The following inclusion criteria were used: (1) age ≥ 50 years; (2) confirmation of PCV with indocyanine angiography (ICG-A); (3) ≥ 12-month history of anti-VEGF monotherapy prior to PDT; (4) minimum follow-up period of 18 months after PDT. Eyes with concurrent visually impairing ocular diseases such as glaucoma, retinal vessel occlusion, diabetic retinopathy, epiretinal membrane, uveitis, and retinal detachment were excluded.

### Patient assessment

At initial presentation and during follow-up visits, the following examinations were performed on all patients: best-corrected visual acuity (BCVA) using early treatment diabetic retinopathy study (ETDRS) letters, slit-lamp biomicroscopy, optical coherence tomography (OCT) imaging (Topcon 3D OCT-2000, Topcon Corporation, Tokyo, Japan; or Spectralis, Heidelberg Engineering, Heidelberg, Germany). OCT angiography was performed prior to starting intravitreal anti-VEGF treatments. ICG-A was not performed routinely from the outset; instead, it was only performed in patients with clinical suspicion of PCV, usually after ≥ 12 months of poor visual or anatomical response to regular anti-VEGF injections. Diagnostic criteria of PCV were based on the presence of angiographic features described by the EVEREST study group [23].

### Treatments

All eyes received anti-VEGF monotherapy as first-line treatment in the presence of subretinal (SRF) or intraretinal fluid (IRF) or sub-macular haemorrhage. Anti-VEGF agents administered included ranibizumab (IVR, 0.5 mg in 0.05 mL) or aflibercept (IVA, 2 mg in 0.05 mL). All eyes had 3 consecutive monthly intravitreal anti-VEGF injections followed by maintenance regimens comprised of fixed dosing and treat and extend (T&E). Those patients who had commenced IVR as the first-line anti-VEGF agent but later switched to IVA due to sub-optimal response were stratified into a separate group (IVS). Refractoriness to ranibizumab was defined as persistent SRF/IRF and worsening BCVA despite patients receiving fixed monthly injections. Once PCV was diagnosed, patients were offered PDT as a “rescue or add-on” treatment. PDT was performed with verteporfin (Visudyne, Novartis, Basel, Switzerland) according to the protocol of the EVEREST II study [14].

All patients were treatment-naïve prior to receiving the treatments described and analysed in this study. Clinical assessments and OCT described above were performed at each follow-up visit. Re-treatment with anti-VEGF agents following PDT was indicated for the following reasons: (1) to stabilise vision and increase treatment interval in eyes that had achieved polyp regression; (2) to prevent sub-macular haemorrhages; (3) to resolve recurrent or persistent SRF/IRF. Ultimately, the decision on re-treatment at each visit, follow-up intervals, switch from one agent to another, and treatment modality was at the managing retinal specialist’s discretion.

### Outcome measures

Baseline characteristics and therapeutic outcomes were compared between the groups. The primary outcomes were mean change in BCVA and anti-VEGF treatment burden. These factors in the pre-PDT (from initial presentation to the day of PDT) and post-PDT (from the day of PDT to 6-, 12-, 18-, 24-, 30-, and 36-month follow-ups) were evaluated. Other parameters evaluated included the proportion of eyes gaining ≧10 letters, losing ≧5 letters, and attaining BCVA ≥ 70 letters, the proportion of eyes achieving an injection interval ≧12 weeks (including PRN and no more injection required), and the percentage of eyes accomplishing “dry macular” (defined as lack of exudative changes including no IRF/SRF on OCT). A last observation carried forward (LOCF) approach was performed in the present study to address attrition bias.

### Statistical analysis

Statistical analysis was performed using SPSS (V.27, IBM, New York, NY, USA). The normality of data was evaluated with histograms and Kolmogorov–Smirnov/Shapiro–Wilk tests. Wilcoxon signed-rank test and Kruskal–Wallis test was used for analysing nonparametric continuous variables, paired *t*-test, and one-way analysis of variance (ANOVA) with or without post hoc analysis was used to analyse parametric continuous variables, and chi-square or Fisher’s exact test were used for categorical data. *P*-values < 0.05 were considered significant.

## Results

A total of 51 PCV cases treated with combined therapy of anti-VEGF intravitreal injection and PDT meeting the inclusion criteria were identified. Five cases were excluded due to concurrent visually impairing ocular diseases and 2 cases were excluded as they had less than 18-month follow-ups. Forty-four eyes were included in the final analysis. Baseline characteristics of the included patients are shown in Table 1. There were no significant differences in demographic characteristics between the three groups except for age – patients in the IVA group were marginally younger compared to the IVR group (73.4 vs 79.7, *p* = 0.038).

**Table 1:** Baseline characteristics of the eyes included in the analysis

	IVR		IVS		IVA	
Eyes, *n*	7		19		18	
Age (mean ± SD)	79.7 (3.6)		78.3 (4.9)		73.2 (8.8)*	
Sex (male)	1	14.3%	6	31.6%	4	22.2%
Laterality (right eye)	3	42.9%	11	57.9%	5	27.8%
Time to PDT (months, mean ± SD)	17.9 (7.4)		33.7 (24.5)		23.1 (14.3)	
Ethnicity						
Caucasian	6	85.7%	17	89.5%	16	88.9%
Afro-Caribbean	0		2	10.5%	1	5.5%
South Asian	1	14.3%	0		1	5.5%
OCT features at baseline, *n*						
SRF	7	100.0%	19	100.0%	17	94.4%
IRF	3	42.9%	7	36.8%	3	16.7%
PED	7	100.0%	19	100.0%	18	100.0%
BCVA at presentation (mean ± SD)	61.3 (7.2)		62.3 (11.2)		63.6 (14.6)	
BCVA on the day of PDT (mean ± SD)	68.9 (4.3)		57.9 (13)		55.5 (14.3)	
Total number of injections pre-PDT (mean ± SD)	9.0 (3.2)		8.9 (2.0)		10.0 (2.2)	

*IVR*, intravitreal ranibizumab; *IVS*, switch from intravitreal ranibizumab to aflibercept; *IVA*, intravitreal aflibercept; *SD*, standard deviation; *PDT*, photodynamic therapy; *BCVA*, best corrected visual acuity

^*^Significantly different based on Games–Howell post hoc analysis

The mean (± standard deviation) time interval from initial presentation to the day of PDT (pre-PDT period) was 17.9 (7.4), 33.7 (24.5), and 23.1 (14.3) months for the IVR, IVS, and IVA groups, respectively (*p* = 0.147). The mean number of injections per year during the pre-PDT period was not significantly different amongst the three groups (IVR: 9.0 ± 3.2; IVS: 8.9 ± 2.0; IVA: 10.0 ± 2.2; *p* = 0.293). During this period, all the eyes in the IVR group were on a fixed-dose 4-weekly intravitreal regimen; in the IVS group, 16 (84.2%) eyes were on the 4-weekly regimen, 3 (15.8%) on a 6-weekly regimen; whereas in the IVA group, 13 (72.2%), and 3 (16.7%) eyes were on 4- and 6-weekly regimens, respectively, and 1 (5.6%) eye each was on 8- and 12-weekly regimens.

**Fig. 1:**
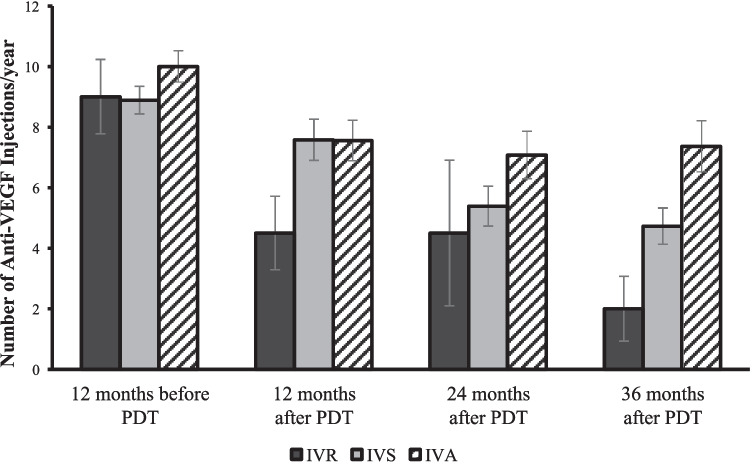
The number of anti-VEGF injections required per year (mean ± standard error of the mean) 12 months before PDT, and at 12 months, 24 months, and 36 months after PDT. The mean number of anti-VEGF injections required over 12, 24, and 36 months after PDT were all significantly less than that over the 12 months before PDT in all three groups (IVR: *p* = 0.017, 0.005, 0.001, respectively; IVS: *p* = 0.147, 0.001, 0.001, respectively; IVA: *p* = 0.005, 0.001, 0.001, respectively)

After PDT, all three groups required significantly less injections at different time points of follow-up than before PDT and showed a downward trend (Fig. 1). The mean number of injections required by eyes in the IVR group were significantly fewer than the IVS (*p* = 0.019) and IVA (*p* = 0.021) groups at 12-month follow-up, but the overall burdens were similar at the longer-term 24- and 36-month time points (*p* = 0.051 and 0.056, respectively). At final follow-up, 3 eyes (42.9%) in the IVR group were on a T&E regimen, 4 eyes (57.1%) were on a pro re nata (PRN); in the IVS group, 1 eye (5.3%) was on the fixed 4-weekly regimen, 15 eyes (78.9%) were on T&E, 3 eyes (15.7%) were on PRN; whilst in the IVA group, 4 eyes (22.2%) and 2 eyes (11.1%) were on fixed 4- and 6-weekly regimen, respectively, and 11 eyes (61.1%) were on T&E, and 1 (5.6%) was on PRN.

At the initial presentation, there were no statically significant differences among the three groups in baseline BCVA (*p* = 0.906). Before receiving ‘rescue’ PDT (pre-PDT period), patients were treated with intravitreal anti-VEGF monotherapy. During this pre-PDT period, the mean BCVA on the day of PDT was significantly poorer compared to baseline at initial presentation in all three groups, despite regular intravitreal injections (IVR: *p* = 0.006**;** IVS: *p* = 0.050; IVA: *p* = 0.020). The BCVA on the day of PDT did not differ significantly amongst the three groups (*p* = 0.070). Eyes in the IVR group gained a mean of 7.6 (4.8) letters, whilst the IVS and IVA groups lost 4.4 (9.4) and 8.1 (13.3) letters, respectively (*p* = 0.009). Significantly higher numbers of eyes in the IVR group gained ≥ 10 letters (IVR: 3 eyes [42.9%]; IVS: 0 eyes; IVA: 2 eyes [11.1%]; *p* = 0.009), whilst greater proportion of eyes in the IVA group lost ≥ 5 letters (IVR: 0 eyes; IVS: 8 eyes [42.1%]; IVA: 11 eyes [61.1%]; *p* = 0.021).

**Fig. 2:**
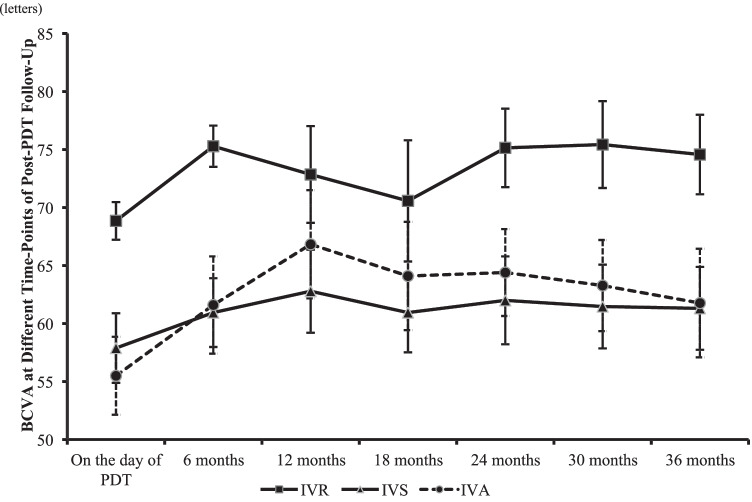
Mean BCVA in ETDRS letters (mean ± standard error of the mean) of the three groups on the day of PDT and at 6-, 12-, 18-, 24-, 30-, and 36-month follow-up after PDT. BCVA improved in all groups at different time points compared to BCVA on the day of PDT, but the improvements were not statistically significant in all time-point after 12-months of PDT (the *P*-values between the BCVA on the day of PDT and each time point were: IVR: 0.01, 0.308, 0.711, 0.096, 0.087, 0.33; IVS: 0.029, 0.006, 0.047, 0.017, 0.035, 0.042; IVA: 0.044, 0.010, 0.033, 0.02, 0.065, 0.112)

**Fig. 3:**
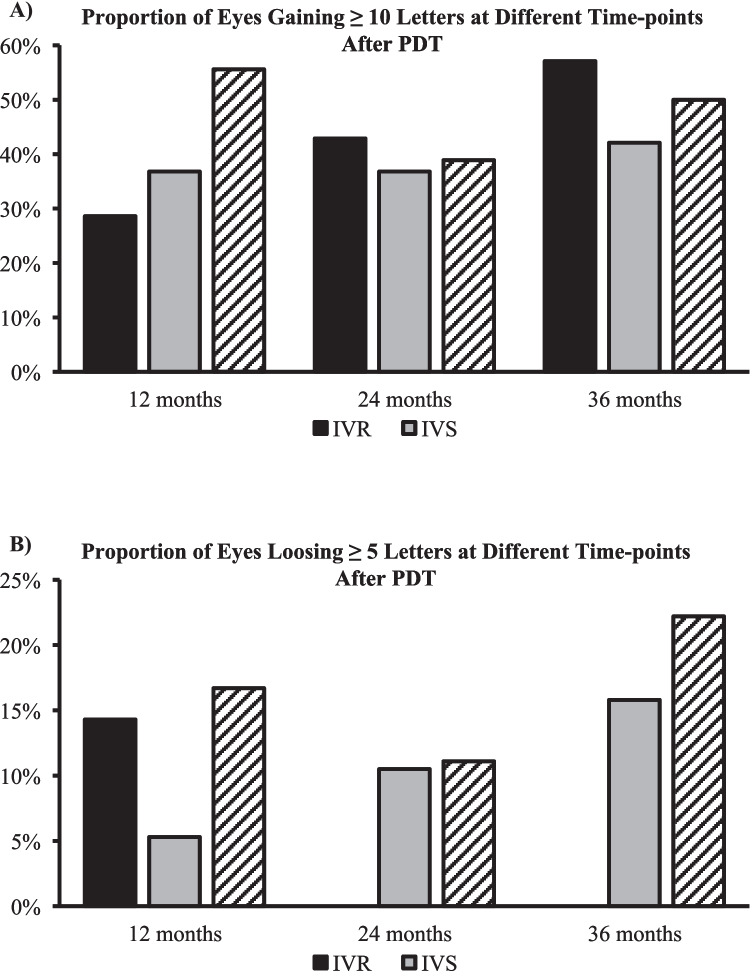
**A** The proportion of eyes with BCVA gain of ≥ 10 EDTRS letters at 12-, 24-, and 36-month follow-up after PDT (*p* = 0.360, 0.961, 0.768). **B** The proportion of eyes with BCVA dropping by ≥ 5 EDTRS letters at 12-, 24-, and 36-month follow-up after PDT (*p* = 0.532, 0.658, 0.394)

Compared to the BCVA on the day of PDT, eyes in all three groups experienced improved visual acuity at 6-month, 12-month, 18-month, 24-month, 30-month, and 36-month follow-ups after PDT (Fig. 2). There were no significant differences observed in BCVA throughout the post-PDT follow-up period between the three groups of eyes (*p* = 0.067, 0.400, 0.445. 0.073, 0.074, 0.137 at 6, 12, 18, 24, 30, and 36 months, respectively). At the final visit after PDT, the mean VA improvement in letter scores from the day of PDT was similar across the three groups (IVR: 5.7 ± 7.8; IVS: 2.5 ± 12.1; IVA: 10.2 ± 16.7; *p* = 0.247). Furthermore, the proportion of eyes gaining ≥ 10 letters and losing ≥ 5 letters were comparable between the groups (Fig. 3).

**Fig. 4:**
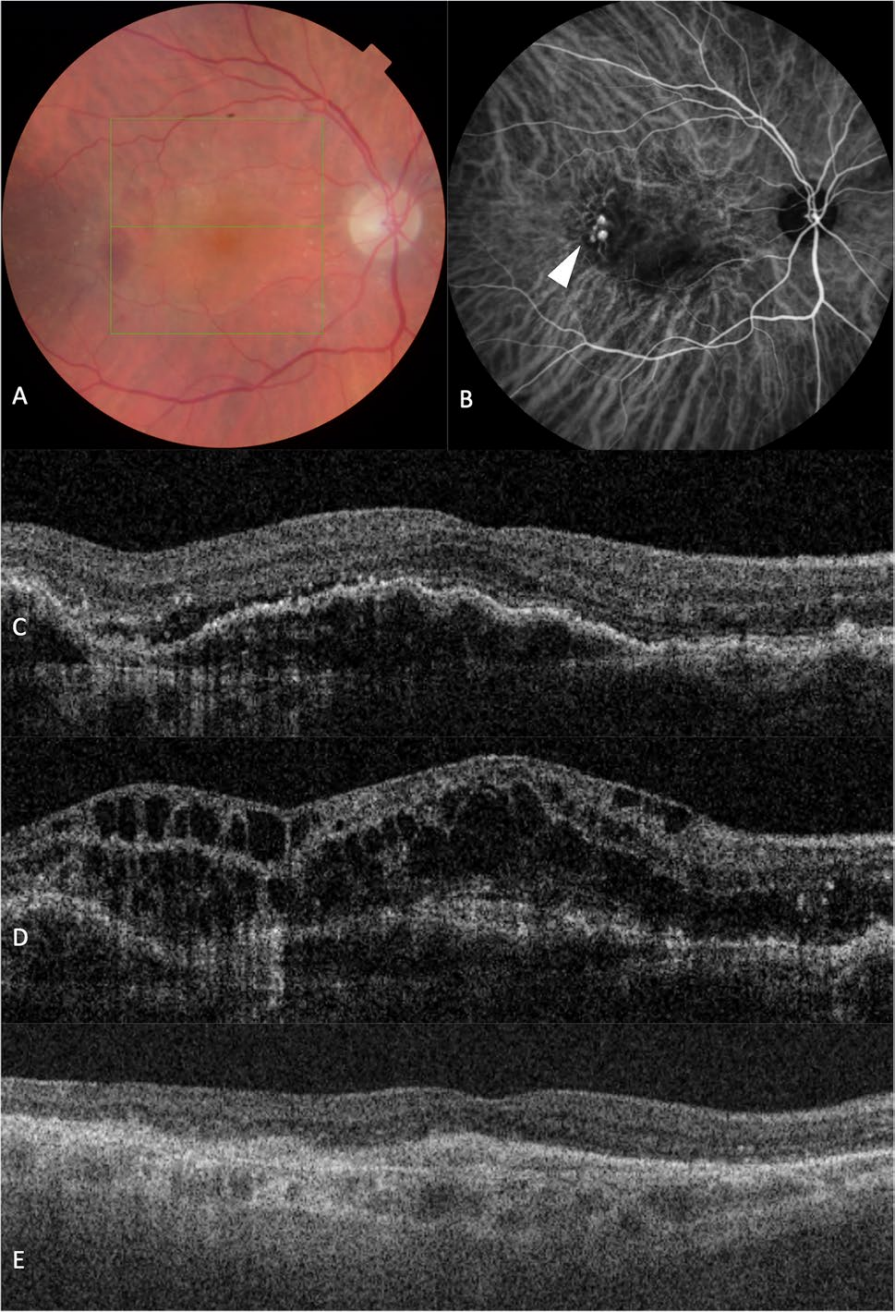
A case of a female patient with persistent disease activity despite 4-weekly IVA monotherapy. Active polyps were only identified on ICG-A after 33 anti-VEGF injections. Substantial response post-PDT was observed with significant reduction in re-treatment burden (currently on T&E regimen to 8-weekly) and resolution of retinal fluid, though BCVA did not improve. **A** Colour fundus photograph at initial presentation shows submacular haemorrhage and the reddish-orange polypoidal lesion. **B** Mid-phase ICG-A reveals active polyps (arrowhead) and abnormal branching vascular network. **C** OCT at initial presentation exhibits SRF and PED. The patient was initially diagnosed with n-AMD and started with IVA monotherapy. **D** Baseline OCT before PDT shows persistent and worsening SRF, PED, and IRF. **E** OCT at 6 months after PDT shows resolution of SRF and IRF, though there is evidence of structural damage with subretinal fibrosis due to delay in ICG-A

At the final visit, “dry macular” (defined as the absence of SRF/IRF/sub-macular haemorrhage) was accomplished by 5 eyes (71.4%) in the IVR group, 9 eyes (47.4%) in the IVS group, and 6 eyes (33.3%) in the IVA group, with no significant differences across the cohorts (*p* = 0.223); whilst PED height was reduced in 6 (85.7%), 12 (63.2%), and 7 (38.9%) eyes from the IVR, IVS, and IVA groups (*p* = 0.080). Figure 4 shows a representative case.

## Discussion

To the best of our knowledge, this is the first study that compared the long-term visual outcomes and re-treatment burden of patients with PCV who had received PDT combined with IVR to those who had received PDT combined with IVA in a European cohort of patients. This retrospective analysis demonstrated that combined PDT with either IVR, IVA, and the switch from IVR to IVA, were all able to reduce intravitreal re-treatment burden and improve visual outcomes, and the treatment outcomes were not significantly different between the three groups.

To date, several studies have demonstrated the therapeutic efficacy of intravitreal aflibercept and ranibizumab as monotherapy for PCV [24, 25]. However, there have been limited reports focusing on comparing the therapeutic effects of the two agents for PCV in Caucasian patients. Although the main aim of the present study was to compare the treatment outcomes of combined therapy of PDT with different intravitreal anti-VEGF agents, we also sought to present the real-world results of different anti-VEGF agents as monotherapy. During the pre-PDT period of the present study, the mean BCVA of eyes receiving IVR monotherapy had improved by 7.6 letters, whilst the visual acuity worsened in eyes that had switched from IVR to IVA and those which had received IVA. In addition, a higher proportion of eyes in the IVR group improved BCVA by more than 10 letters, whilst less of them experienced worsened visual acuity. On the contrary, Cho et al. found that BCVA improved by 8.5 and 9.5 letters in IVR and IVA-treated patients, and 26.7 and 31.6% of IVR and IVA-treated patients experience BCVA improvement of more than 15 letters after 12 months of injections in a Korean cohort of PCV patients [26]. The follow-up period since the initiation of anti-VEGF injections was much longer in the present study, and it was conducted on a Caucasian-majority European cohort. These factors may contribute to the discrepancies in treatment outcomes.

Ranibizumab was one of the most widely used anti-VEGF agents and has been demonstrated to be efficacious for treating PCV [13, 27]. However, some PCV cases may not respond to ranibizumab adequately [28–30]. Since the introduction of the newer aflibercept, several studies have reported that switching from IVR to IVA is effective for improving visual acuity, resolving exudative lesions, and regressing polypoidal lesions in eyes with PCV refractory to ranibizumab [31–34]. The difference in molecular mechanism and pharmacological mode of action between the two anti-VEGF agents were hypothesised to be the reason for the improved outcome after switching [35]. In the present study, the majority of eyes (73.1%) started with receiving IVR and switched to IVA after at least 12-months of inadequate response to ranibizumab, which was markedly higher than the switch rate (39.5%) reported previously [33]. Moreover, contrary to the results reported in other studies, switching to IVA did not appear to improve the outcome of PCV refractory of IVR in our cohort, as mean BCVA continued to decline and no eyes managed to gain ≥ 10 letters.

Frequent re-treatment with anti-VEGF injections represents a significant burden on patients, physicians, and society, especially in regions where universal coverage for healthcare is not available. Hence it may be useful to analyse if one anti-VEGF agent has superior efficacy over the other. The current study showed that all eyes required significantly less anti-VEGF injections per year during the 3-year follow-up period after PDT than before PDT, and the re-treatment burden was not significantly different 12 months after PDT, regardless of the agents used. Multiple studies have reported less re-treatment requirements in eyes that had received IVA in combination with PDT than those that had been treated with IVR and PDT [21, 22]. On the other hand, Kikushima et al. showed no differences in the re-treatment exposure between the two groups [16]. The results of this comparative analysis are mixed but our data demonstrates that the choice of anti-VEGF agents does not significantly impact the re-treatment burden of combination therapy in PCV.

Similar to the results reported by other authors [21, 22], the present study also found that the BCVA in eyes treated with either IVR or IVA was significantly improved after receiving PDT, and further demonstrated that the visual improvements were able to sustain over 36-months of follow-up. Weng et al. and Ito et al. reported no differences in the improvement of BCVA between eyes receiving combination therapy of PDT with IVR or IVA 12-months after treatment [21, 22], whilst Kikushima et al. demonstrated significantly better BCVA in eyes that had received PDT in combination with IVA, than those that were treated with IVR at 18- and 24-month follow-up [16]. Although the patients treated with PDT and IVR in our European cohort achieved better final BCVA at 36-month follow-up, the differences were not statistically significant compared to other groups. It is noteworthy that the pre-PDT baseline BCVA of the IVS and IVA groups were worse than the IVR group, which may explain the observed difference in post-PDT visual outcomes. The long-term visual and anatomical outcomes of our PCV cohort were not statistically different between the choice of anti-VEGF agents used in combination with PDT.

There is currently no comparative data on the treatment outcomes of PDT after switching anti-VEGF agents from IVR to IVA in European patients. Our real-world data suggested that switching to IVA in eyes refractory to IVR would not necessarily produce better results. In fact, eyes in the IVS group of the present study had the worst final visual outcome. Although there are some evidences that support switching to IVA in eyes refractory to IVR alone [31, 33], adding PDT instead of changing anti-VEGF agents may be able to offer more substantial benefit owing to its superior ability in inducing complete polyp regression [36]. Besides, the structural damage to retina and choroid during a prolonged period of sub-optimal responses to anti-VEGF monotherapies may cause irreversible visual loss. Administering PDT promptly in eyes refractory to any anti-VEGF agent is likely a better option in reducing re-treatment burden and achieving improved visual results than trialling with another anti-VEGF agent. Although, with the newer agents on the horizon, anti-VEGF monotherapy may be a potential option for PCV in the future, as early short-term data for these agents have demonstrated promising outcomes as monotherapies [37, 38].

PCV has been described as the most common form of n-AMD in East Asian population [39], whilst the prevalence of PCV in Caucasian individuals was estimated to be much lower at 8.7% [40]. In the present European cohort, 88.6% of the patients were Caucasian individuals. This finding suggests that the prevalence of PCV in Caucasian is probably underestimated, therefore in eyes with presumed n-AMD refractory to anti-VEGF monotherapy, high index of suspicion for PCV is needed so prompt PDT can be performed to enable polyp regression and stabilise disease, especially as ICG-A is not performed routinely [8].

This study is limited by the retrospective nature of analysis and small sample size. As patients were not randomised and assigned to specific anti-VEGF agents before treatments started, the comparability of our study to larger prospective analysis might be hampered. Secondly, all patients presented to our centre with suspected n-AMD underwent OCT and OCT-angiography at the initial consultation, but not ICG-A. The lack of mandatory ICG-A unsurprisingly led to delay in diagnosis, which was the primary reason for the use of anti-VEGF monotherapy as a first-line treatment. Moreover, ICG-A was not performed during follow-up after PDT, therefore polyp regression could not be assessed. The diagnostic pathways described here does represent real-world practices in majority of ophthalmology services in the UK, especially in centres where ICG-A is not routinely available [41]. Recent expert consensus on OCT-based diagnostic criteria for PCV could potentially allow for prompt recognition of PCV without the need of the more invasive and time-consuming ICG-A [42]. Finally, PDT was performed after a prolonged period of sub-optimal responses to anti-VEGF monotherapy in the present study; thus, cautions should be taken when drawing a direct comparison with the outcomes of other studies as patients enrolled in most existing reports were treatment naïve. Since May 2020, the global supply of verteporfin has been interrupted due to reduced manufacturing capabilities [43]. Whilst we advocate the prompt administration of PDT to induce polyp regression and stabilise disease, due to verteporfin shortage, some patients may experience a significant delay in receiving PDT [44]. Until the supply crisis is resolved, anti-VEGF monotherapy might be the only option for controlling PCV in these patients, and clinicians need to remain vigilant due to the risk of recurrent submacular haemorrhage in between injections. Further prospective analyses with larger sample sizes are thus required to determine the optimal choice of anti-VEGF agents to combine with PDT for the treatment of PCV.

In summary, this is the first study presenting the long-term real-world outcomes of combination therapy of PDT with IVR and IVA in a Caucasian-majority European cohort of patients. Both IVR and IVA combined with PDT were effective treatments for PCV. There were no significant differences in the final visual outcomes and re-treatment burden between the groups. The administration of PDT is likely more critical for the effective management of PCV than the choice of anti-VEGF agents. Furthermore, in eyes refractory to IVR, performing PDT promptly may be more beneficial than switching to IVA.
